# Musicians Show Improved Speech Segregation in Competitive, Multi-Talker Cocktail Party Scenarios

**DOI:** 10.3389/fpsyg.2020.01927

**Published:** 2020-08-18

**Authors:** Gavin M. Bidelman, Jessica Yoo

**Affiliations:** ^1^Institute for Intelligent Systems, University of Memphis, Memphis, TN, United States; ^2^School of Communication Sciences and Disorders, University of Memphis, Memphis, TN, United States; ^3^Department of Anatomy and Neurobiology, University of Tennessee Health Sciences Center, Memphis, TN, United States

**Keywords:** acoustic scene analysis, stream segregation, experience-dependent plasticity, musical training, speech-in-noise perception

## Abstract

Studies suggest that long-term music experience enhances the brain’s ability to segregate speech from noise. Musicians’ “speech-in-noise (SIN) benefit” is based largely on perception from simple figure-ground tasks rather than competitive, multi-talker scenarios that offer realistic spatial cues for segregation and engage binaural processing. We aimed to investigate whether musicians show perceptual advantages in cocktail party speech segregation in a competitive, multi-talker environment. We used the coordinate response measure (CRM) paradigm to measure speech recognition and localization performance in musicians vs. non-musicians in a simulated 3D cocktail party environment conducted in an anechoic chamber. Speech was delivered through a 16-channel speaker array distributed around the horizontal soundfield surrounding the listener. Participants recalled the color, number, and perceived location of target callsign sentences. We manipulated task difficulty by varying the number of additional maskers presented at other spatial locations in the horizontal soundfield (0–1–2–3–4–6–8 multi-talkers). Musicians obtained faster and better speech recognition amidst up to around eight simultaneous talkers and showed less noise-related decline in performance with increasing interferers than their non-musician peers. Correlations revealed associations between listeners’ years of musical training and CRM recognition and working memory. However, better working memory correlated with better speech streaming. Basic (QuickSIN) but not more complex (speech streaming) SIN processing was still predicted by music training after controlling for working memory. Our findings confirm a relationship between musicianship and naturalistic cocktail party speech streaming but also suggest that cognitive factors at least partially drive musicians’ SIN advantage.

## Introduction

In naturalistic sound environments, the auditory system must extract target speech and simultaneously filter out extraneous sounds for effective communication – the classic “cocktail-party problem” (Cherry, 1953; Bregman, 1978; Yost, 1997). Auditory stream segregation refers to the ability to identify and localize important auditory objects (cf. sources) in the soundscape. The ability to stream is highly relevant to both speech and music perception, e.g., communicating in a noisy restaurant or following a symphonic melody (Fujioka et al., 2005; Shamma et al., 2011). Successful streaming depends on Gestalt-like processing (Holmes and Griffiths, 2019) but also hearing out important acoustic cues including harmonic structure, spatial location, and onset asynchrony, all of which can promote or deny perceptual segregation (Carlyon, 2004; Alain, 2007).

Several experiential (e.g., language expertise and musical training) and cognitive factors [e.g., attention and working memory (WM)] have been shown to influence auditory stream segregation (Broadbent, 1958; Fujioka et al., 2005; Singh et al., 2008; Zendel and Alain, 2009; Ruggles and Shinn-Cunningham, 2011; Bidelman and Dexter, 2015; Zendel et al., 2015). Musicianship, in particular, has been associated with widespread perceptual–cognitive enhancements that help the brain resolve the cocktail party problem. Indeed, musically savvy individuals are highly sensitive to changes in auditory space (Munte et al., 2001) and tracking voice pitch (Bidelman et al., 2011) and are better than their non-musician peers at detecting inharmonicity in sound mixtures (Zendel and Alain, 2009). These features are prominent cues that signal the presence of multiple acoustic sources (Popham et al., 2018), and musicians excel at these skills.

A widely reported yet controversial benefit of music engagement is the so-called “musician advantage” in speech-in-noise (SIN) processing (for review, see Coffey et al., 2017). Several studies demonstrate that musicians outperform non-musicians in figure-ground perception, as measured in a variety of degraded speech recognition tasks (Bidelman and Krishnan, 2010; Parbery-Clark et al., 2011; Swaminathan et al., 2015; Anaya et al., 2016; Clayton et al., 2016; Brown et al., 2017; Deroche et al., 2017; Du and Zatorre, 2017; Mankel and Bidelman, 2018; Torppa et al., 2018; Yoo and Bidelman, 2019). Amateur musicians (∼10 years training) are better at identifying and discriminating target speech amidst acoustic interferences including reverberation (Bidelman and Krishnan, 2010) and noise babble (Parbery-Clark et al., 2009a). In standardized (audiological) measures of SIN perception [e.g., Hearing in Noise Test (HINT) and QuickSIN test] (Nilsson et al., 1994; Killion et al., 2004), musicians also tolerate ∼1 dB more noise than their non-musician peers during degraded speech recognition (Parbery-Clark et al., 2009b; Zendel and Alain, 2012; Mankel and Bidelman, 2018; Yoo and Bidelman, 2019). Similar results transfer to non-speech sounds (Fuller et al., 2014; Başkent et al., 2018). Still, not all studies report a positive effect, and some fail to find a musician advantage even on identical SIN tasks (e.g., QuickSIN and HINT) (Ruggles et al., 2014; Boebinger et al., 2015; Madsen et al., 2017; Yeend et al., 2017; Escobar et al., 2020). The failure to replicate could be due to the small nature of this effect and/or, as we have previously suggested, unmeasured differences in music aptitude even among self-reported musicians that confer perceptual gains in SIN processing (Bidelman and Mankel, 2019). Musicians’ SIN benefits are also more apparent in older adults (Zendel and Alain, 2012), so the predominance of studies on young adults may not be representative of music-related SIN benefits. Nevertheless, a handful of studies suggest (albeit equivocally) that music training might improve the ability to segregate multiple sound streams in relation to cocktail party listening.

To date, prior studies on the effects of long-term music experience and SIN processing have focused on simple headphone-based figure-ground tasks rather than stream segregation or true cocktail party listening, *per se* (but see Madsen et al., 2019). We know that musicians are less affected by informational masking (Oxenham and Shera, 2003; Swaminathan et al., 2015; Yoo and Bidelman, 2019) and that their SIN advantages are stronger when targets and maskers are both speech (Swaminathan et al., 2015; Yoo and Bidelman, 2019). For example, using a task decomposition strategy (e.g., Coffey et al., 2017), we recently examined musicians’ performance in a number of speech (and non-speech) masking tasks in order to identify conditions under which musicians show listening benefits in adverse acoustic conditions (Yoo and Bidelman, 2019). We found that musicians excelled in SIN perception but most notably for speech-on-speech masking conditions, i.e., those containing substantial linguistic interference and higher degrees of information masking (see also Swaminathan et al., 2015). Thus, the “musician SIN benefit” depends largely on task structure (Yoo and Bidelman, 2019). Moreover, cocktail party listening draws upon general cognitive faculties (e.g., memory and attention), and musicians are known to differ from non-musicians in the domains of WM (Bugos et al., 2007; Bidelman et al., 2013; Yoo and Bidelman, 2019), attention (Strait et al., 2010; Strait and Kraus, 2011; Sares et al., 2018; Medina and Barraza, 2019; Yoo and Bidelman, 2019), and executive functioning (Bugos et al., 2007; Bialystok and Depape, 2009; Moreno et al., 2011; for review, see Moreno and Bidelman, 2014; Zuk et al., 2014; Lerousseau et al., 2020).

While we and others have shown musicians are unusually good at parsing simultaneous speech (at least diotically/monaurally), it remains unclear if these benefits translate to more naturalistic acoustic environments that offer spatial cues for segregation and engage binaural processing. Spatialization is an important acoustic cue listeners exploit to parse multiple talkers and aid speech recognition in normal cocktail party scenarios (Nelson et al., 1998). This realistic component of normal scene analysis is not testable using conventional SIN tests, limiting ecological validity of previous work. Moreover, given evidence that musicianship might engender enhanced cognitive functioning (Schellenberg, 2005; Moreno and Bidelman, 2014), we were interested to test the degree to which musicians’ cocktail party benefits might be explained by domain general skills.

In light of the equivocal nature of musicians’ SIN benefit(s), our aim was to assess whether they show perceptual advantages in speech segregation in a competitive, multi-talker environment, thereby confirming their putative SIN benefits but extending them to more ecological “cocktail party” scenarios. To this end, we measured speech streaming abilities in musicians and non-musicians using a realistic, 3-D cocktail party environment. The study was conducted in the unique setting of an anechoic chamber with surround sound stimulus presentation. We hypothesized musicians would show more accurate performance than non-musicians in cocktail party speech recognition and localization tasks, extending prior results from laboratory-based SIN tasks. We further expected to find associations among cognitive factors such as attention and WM with stream-segregation performance (e.g., Yoo and Bidelman, 2019). This would suggest a role of cognitive factors in partially driving musicians’ cocktail party advantages.

## Materials and Methods

### Participants

Young (*N* = 28, age range: 19–33 years), normal-hearing adults were recruited for the study. The sample was divided into two groups based on self-reported music experience. Fourteen musicians (M; nine females and five males) had at least 9 years of continuous training (15.07 ± 4.14 years) on a musical instrument starting before age 10 (7.2 ± 2.49 years). Fourteen non-musicians (NM; 10 females and 4 males) were those with ≤4 years (0.89 ± 1.23 years) of lifetime music training on any combination of instruments. Instruments included piano (2), percussion (3), oboe (1), tuba (1), voice (1), saxophone (1), trumpet (1), French horn (2), guitar/bass (1), and clarinet (1). All were currently active in playing their instrument in an ensemble or private setting. All showed normal-hearing sensitivity (puretone audiometric thresholds ≤ 25 dB HL; 250 to 8,000 Hz) and had no previous history of brain injury or psychiatric problems. Non-native speakers perform worse on SIN tasks than their native-speaking peers. Thus, all participants were required to be native speakers of English (Rogers et al., 2006; Bidelman and Dexter, 2015). The two groups were otherwise matched in age (*t*_26_ = −0.43, *p* = 0.67), right-handedness as measured by the Edinburgh Handedness inventory (Oldfield, 1971; *t*_26_ = 1.84, *p* = 0.08), gender (Fisher’s exact test: *p* = 1.0), formal education (*t*_26_ = 0.51, *p* = 0.62), and socioeconomic status (*t*_26_ = 0.48, *p* = 0.64), scored based on the highest level of parental education: 1 (high school without diploma or GED)–6 (doctoral degree) (Norton et al., 2005; Mankel and Bidelman, 2018). Each gave written informed consent in accordance with a protocol approved by the University of Memphis Institutional Review Board.

### Stimuli and Task Paradigms

We measured naturalistic cocktail party listening skills via a sentence-on-sentence speech recognition task (Bolia et al., 2000) conducted in a 3D spatial field (described below). As a comparison to normed SIN measures, we also measured QuickSIN scores (Killion et al., 2004), which have previously revealed musician advantages in SIN perception (Zendel and Alain, 2012; Mankel and Bidelman, 2018; Yoo and Bidelman, 2019). Domain general cognitive skills [i.e., fluid intelligence (IQ), WM, and sustained attention] were evaluated using Raven’s progressive matrices (Raven et al., 1998), backwards digit span (Wechsler et al., 2008), and the Sustained Attention to Response Task (SART) (Robertson et al., 1997), respectively.

#### Speech Streaming Task

**FIGURE 1:**
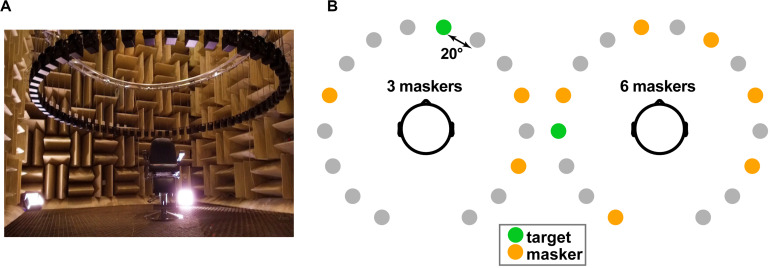
Cocktail party streaming task. **(A)** Participants were seated in the center of a 16-channel speaker array within an anechoic chamber. Speaker heights were positioned at ear level (∼130 cm) during the task with a radial distance of 160 cm to the center of the head and speaker-to-speaker distance of ∼20°. **(B)** Example stimulus presentation (three- and six-talker conditions). Participants were asked to recall the color, number, and perceived location of target callsign sentences from the coordinate response measure (CRM) corpus (Bolia et al., 2000). Target location was varied randomly from trial to trial and occurred simultaneous with between zero and eight concurrent masking talkers.

We measured speech recognition and localization performance in a simulated multi-talker cocktail party environment within the University of Memphis Anechoic Chamber (Figure 1A)^1^. A 16-channel circular speaker array was positioned vertically 130 cm above the mesh floor of the anechoic chamber (approximately ear height). Each speaker had a radial distance of 160 cm to the center of the head. Speaker-to-speaker distance was ∼20°. Stimuli were presented at 70 dB SPL (z-weighted, free field), calibrated using a Larson–Davis sound level meter (Model LxT).

We used coordinate response measure (CRM) sentences (Bolia et al., 2000) to measure speech recognition in a multi-talker sounds mixture. CRM sentences contain a different target callsign (Charlie, Ringo, Laker, Hopper, Arrow, Tiger, Eagle, and Baron), color (blue, red, white, and green), and number (1–8) combination embedded in a carrier phrase (e.g., “Ready *Charlie*, go to *blue three* now”). The corpus contained all possible permutations of these callsign–color–number combinations spoken by eight different talkers (male and female). We used CRM sentences as they are sufficiently novel to listeners to avoid familiarity effects that might confound SIN recognition (Johnsrude et al., 2013; Holmes et al., 2018). They are also natural productions that offer a level of control (e.g., similar length and same sentence structure). Participants were cued to the target callsign before each block and were instructed to recall its color–number combination via a sequential button press on the keyboard as fast and accurately as possible (e.g., “b2” = blue–two and “r6” = red–six). We logged both recognition accuracy and reaction times (RTs). RTs were clocked from the end of the stimulus presentation. There were a total of 32 trials per block, repeated twice (i.e., 64 trials per masker condition).

On each trial, listeners heard a mixture of sentences, one of which contained the target callsign and additional CRM sentence(s) that functioned as multi-talker masker(s). Three additional constraints were imposed on sentence selection to avoid unnecessary task confusion: (1) targets were always from the same talker and callsign (within a block); (2) maskers were absent of any callsign, color, and number used in the target phrase (i.e., the callsign’s information was unique among the speech mixture); and (3) target and masker(s) were presented from unique spatial locations (i.e., different speakers). The target speaker/callsign was allowed to vary between blocks but was fixed within block. Males and females were selected randomly. Thus, on average, targets and maskers were 50% male and 50% female. Presentation order and spatial location of the sentences in the 360° soundfield were otherwise selected randomly (Figure 1B).

In separate blocks, we manipulated task difficulty by parametrically varying the number of additional maskers (0, 1, 2, 3, 4, 6, and 8) presented at other spatial locations in the speaker array. We required participants to identify *both* the call color and number of the target callsign phrase to be considered a correct response (chance level = 3.13% = 1/32). It is possible for listeners to localize sound sources even if they cannot identify them (Rakerd et al., 1999). Consequently, after recognition, we had participants indicate the perceived location (azimuth) of the target by clicking on a visual analog of the speaker array displayed on the screen (cf. Figure 1B).

#### QuickSIN

The QuickSIN provided a normed test of SIN reception thresholds. Participants heard six sentences embedded in four-talker noise babble, each containing five keywords. Sentences were presented at 70 dB HL. The signal-to-noise ratio (SNR) decreased parametrically in 5 dB steps from 25 to 0 dB SNR. At each SNR, participants were instructed to repeat the sentence, and correctly recalled keywords were logged. We computed their SNR loss by subtracting the number of recalled target words from 25.5 (i.e., SNR loss = 25.5 - total correct). The QuickSIN was presented binaurally via Sennheiser HD 280 circumaural headphones. Two lists were run, and the second was used in subsequent analysis to avoid familiarization effects (Yoo and Bidelman, 2019).

#### SART

Attention was assessed using the SART (Robertson et al., 1997) implemented in PsychoPy2 (Peirce et al., 2019). Participants rapidly pressed a button for digits (1–9) presented on the computer screen but withheld their response for the digit 3 (i.e., Go/No-Go paradigm). Both correct and incorrect responses were logged, allowing for analysis of omission and commission errors (Van Schie et al., 2012).

#### Digit Span

Backwards digit span was used to assess WM ability. The test consisted of seven questions (each repeated twice). A series of digits was verbally presented to listeners (∼1/s), which varied in sequence length. The length started with two digits (e.g., 2 and 4) and progressively increased to eight digits (e.g., 7, 2, 8, 1, 9, 6, 5, and 3). Participants had to recall the sequence in reverse order. Participants were given 1 point for each correct response. The total score (out of 14) was taken as the individual’s WM capacity.

#### Raven’s Matrices

Raven’s (1998) progressive matrices was used to evaluate non-verbal fluid IQ. Each question contained a 3 × 3 matrix of different abstract patterns and shapes, and participants were instructed to select the missing pattern from one of eight options. Questions became progressively more difficult, which required greater reasoning ability and intellectual capacity. One of two test versions was randomly chosen. They were given 10 min to complete 29 questions. Percent correct scores were recorded.

### Statistical Analysis

Group differences were evaluated for each auditory/cognitive task using independent-samples *t*-tests. Tukey–Kramer adjustments corrected for multiple comparisons. We conducted two-way, mixed-model ANOVAs (group × masker count; subjects = random effect) on speech streaming measures (% accuracy, RTs, and localization error). The control (zero masker) condition was excluded from the ANOVA, though we note that the results were qualitatively similar with or without its inclusion. Dependent measures were log(.) transformed to satisfy homogeneity of variance assumptions necessary for parametric ANOVAs. Pearson correlations assessed (i) the relation between performance on the different speech and cognitive tasks and (ii) whether individuals’ years of music training predicted their perceptual–cognitive skills. Multiple regressions were corrected using the false discovery rate (FDR) (Benjamini and Hochberg, 1995). Effect sizes are reported as $\eta_{\text{p}}^{2}$ for ANOVAs and Cohen’s *d* for *t*-tests.

## Results

**FIGURE 2:**
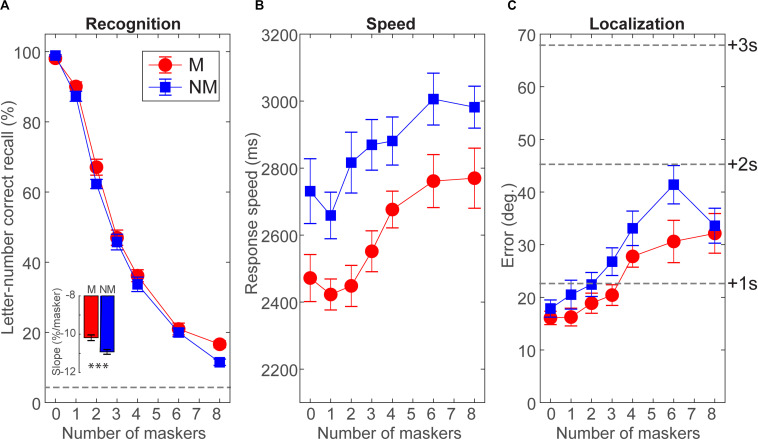
Cocktail party listening is superior in musicians. **(A)** Speech recognition declines with increasing masker counts in both groups, but musicians show less performance decrement up to eight interfering talkers (*inset*). Dotted line = chance performance. **(B)** Musicians show faster (∼200–400 ms) speech recognition speeds than non-musicians. **(C)** Both groups localized correctly identified targets within two speakers (<40° error) with better localization in musicians. Error bars = ± 1 s.e.m.

Group speech streaming performance (i.e.,% accuracy, RTs, and localization error) is shown in Figure 2. Speech recognition expectedly declined from ceiling (M = 98%; NM = 99%) to near-floor (M = 17%; NM = 12%) performance with increasing masker counts from zero (unmasked) to eight multi-talkers. Both groups showed the single largest decrement with two talkers, consistent with prior auditory stream segregation studies (Rosen et al., 2013). Still, both groups showed above-chance recognition even amid eight maskers (all *p*s < 0.0001; *t*-test against 0). Notably, we found a group × masker interaction on target speech recognition accuracy [*F*_(5_, _130)_ = 4.48, *p* = 0.0008, $\eta_{p}^{2}$ = 0.15; Figure 2A]. This interaction was attributable to the change in performance from zero to eight talkers being shallower in musicians compared to non-musicians (Figure 2A, *inset*; *t*_26_ = 3.84, *p* = 0.0007, *d* = 1.45). This suggests that musicians were less challenged by cocktail party speech recognition with an increasing number of interfering talkers.

For speed, we found main effects of group [*F*_(1_, _26)_ = 9.73, *p* = 0.0044, $\eta_{p}^{2}$ = 0.18] and masker count [*F*_(5_, _130)_ = 28.20, *p* < 0.0001, $\eta_{p}^{2}$ = 0.52] on speech recognition RTs (Figure 2B). These data reveal that while decision speeds were predictably slower in more challenging multi-talker scenarios, musicians were faster at streaming target speech across the board.

Localization errors are shown in Figure 2C. Both groups localized targets (correct trials) within about two speakers (<40° error). Localization varied with masker count [*F*_(5_, _130)_ = 21.61, *p* < 0.0001, $\eta_{p}^{2}$ = 0.45], suggesting that target speech segregation worsened with additional talkers. However, musicians showed better localization than non-musicians overall [*F*_(1_, _26)_ = 4.32, *p* = 0.0478, $\eta_{p}^{2}$ = 0.14.

**FIGURE 3:**
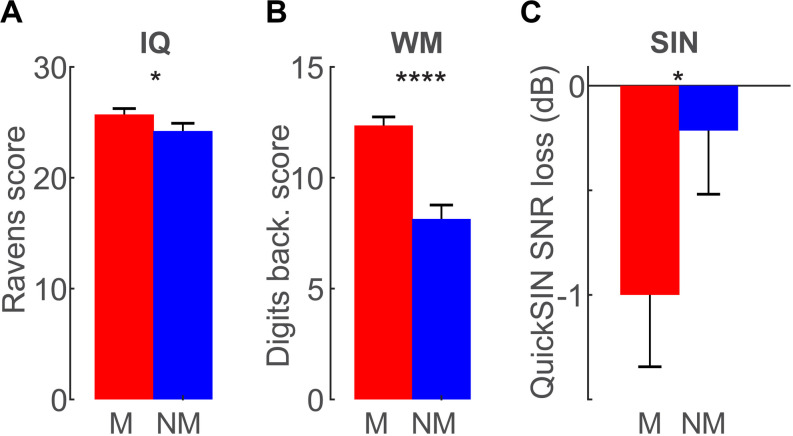
Cognitive skills are superior in musicians. **(A)** Raven’s fluid IQ and **(B)** auditory working memory are enhanced in musicians. **(C)** Musicians also obtain ∼1 dB lower reception thresholds on the QuickSIN test, consistent with the notion of a musician advantage in speech-in-noise (SIN) perception. No group differences were observed in sustained attention (data not shown). Error bars = ± 1 s.e.m. *p < 0.05, ****p < 0.0001.

Group differences in cognitive performance are shown in Figure 3. Replicating prior studies (e.g., Yoo and Bidelman, 2019), we found musician-related advantages in fluid IQ (*t*_26_ = 1.72, *p* = 0.0491, *d* = 0.65; Figure 3A) and backwards WM score (*t*_26_ = 5.72, *p* < 0.0001, *d* = 2.16; Figure 3B). Musicians also outperformed non-musicians by ∼1–2 dB on the QuickSIN test (*t*_26_ = −1.71, *p* = 0.049, *d* = 0.65; Figure 3C), consistent with their superior performance on the speech streaming task (present study) and prior work showing musician benefits in basic SIN perception (Zendel and Alain, 2012; Mankel and Bidelman, 2018; Yoo and Bidelman, 2019). Sustained attention, as measured via the SART, did not differ between groups for either commission (Ms: 35.5% vs. NMs: 29.8%; *p* = 0.41) or omission (Ms: 1.71% vs. NMs: 8.0%; *p* = 0.38) error rates (data not shown). Collectively, these results demonstrate that musicians have better performance than non-musicians in both SIN listening and some general cognitive abilities including IQ and WM.

**FIGURE 4:**
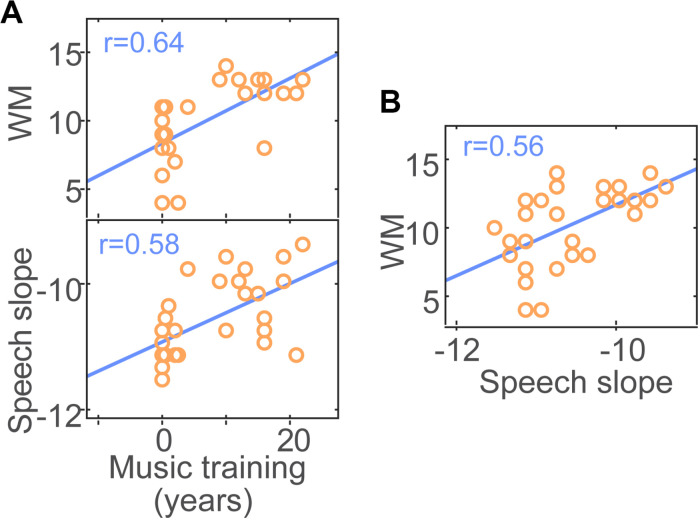
Correlation results. **(A)** Formal music training predicts musicians’ perceptual–cognitive advantages in working memory (WM) and speech streaming at the cocktail party. More extensive music training is associated with better auditory WM and shallower masker-related declines in speech streaming (see Figure 2A, inset). **(B)** Speech streaming is also related to WM; higher WM capacity predicts better cocktail party performance.

We used pairwise correlations to evaluate relations between perceptual and cognitive measures as well as links between musical training and task performance (e.g., Yoo and Bidelman, 2019). Among the family of correlations we assessed (see Supplementary Figure S1 for all 64 bivariate correlations; *p* < 0.05, uncorrected), three survived FDR correction for multiple comparisons: music training was associated with WM and speech streaming performance (Figure 4A). That is, listeners’ years of formal music training predicted better auditory WM scores (*r* = 0.64, *p*_FDR_ = 0.0069) and shallower masker-related declines in speech streaming (*r* = 0.58, *p*_FDR_ = 0.0189). However, we also found that speech streaming correlated with WM such that higher WM capacity predicted better performance at the cocktail party (*r* = 0.56, *p*_FDR_ = 0.0189; Figure 4B). These data suggest that while musicianship is positively associated with improved speech streaming, successful cocktail party listening is at least partially related to cognitive abilities^2^. The association between musical training and SIN processing survived after controlling for WM for the QuickSIN (*r*_partial_ = −0.38, *p* = 0.045) but not speech streaming (*r*_partial_ = −0.34, *p* = 0.08) (cf. Yoo and Bidelman, 2019; Escobar et al., 2020).

## Discussion

By measuring speech recognition in a multi-talker soundscape, we show that trained musicians are superior to their non-musician peers in deciphering speech within a naturalistic cocktail party environment. We found that musicians had faster and better target speech recognition amidst up to almost eight simultaneous talkers and enjoyed less noise-related decline in performance with increasing masker counts relative to musically naïve listeners. These SIN benefits were paralleled in normative measures of figure-ground perception (i.e., QuickSIN test). Our findings confirm and extend prior studies by demonstrating a relationship between musicianship and cocktail party listening skills (stream segregation) but also suggest that cognitive factors may at least partially account for music-related advantages in auditory scene analysis.

Regardless of music background, all listeners showed reduced ability to recognize target speech with increasing talker interferences. Poorer speech recognition with additional talkers is consistent with a reduction in spatial release from masking as more concurrent streams reduce the separability of the target in the soundfield (Pastore and Yost, 2017). More limited performance at higher masker counts is consistent with previous behavioral studies which show that spatial release from masking is effectively limited to fewer than six sound sources (Yost, 2017). Nevertheless, group differences revealed musicians showed smaller masker-related changes in recognition accuracy; trained listeners experienced a 10% decrease in accuracy for additional talkers vs. the 11–12% observed for the untrained group. This small but measurable boost in performance was paralleled in measures of conventional figure-ground SIN perception. We found that musicians had 1–2 dB better speech reception thresholds on the QuickSIN test. While modest, a 1–2 dB benefit in SNR can equate to improvements in speech recognition by as much as 10–15% (Middelweerd et al., 1990), which is comparable to the benefit we find in our cocktail party task. Our findings replicate and extend prior work on the so-called musician advantage for SIN perception (Parbery-Clark et al., 2009b; Zendel and Alain, 2012; Coffey et al., 2017; Mankel and Bidelman, 2018; Yoo and Bidelman, 2019) by demonstrating improved performance in challenging cocktail party speech streaming.

Our findings converge with prior behavioral studies using similar sample sizes (*N* = 20–30) that suggest a musician advantage in spatial release from masking as measured by the improvement in perception with spatially separated vs. co-located speech (Swaminathan et al., 2015; Clayton et al., 2016). However, using a similar paradigm as Swaminathan et al. (2015), but in an anechoic soundfield, Madsen et al. (2019) did not find a musician advantage in streaming performance in their sample of *N* = 64 listeners. However, we note that the evaluation of “cocktail party” listening in all three studies was limited to only a centrally located target (presented in front of the listener) concurrent with two flanking maskers (±15°). In contrast, our design used highly complex multi-talker mixtures (up to eight concurrent talkers) and roved the spatial relation(s) between target and masker(s) in the entire 360° soundfield. Furthermore, our listeners were able to stream using their individualized (natural) head-related transfer functions (HRTFs) rather than simulations as in headphone studies – which limits localization and externalization (cf. Swaminathan et al., 2015). Our data show that musicians outperform non-musicians in these highly ecological cocktail party scenarios at medium to large effect sizes. Collectively, we infer that musician benefits in cocktail party speech perception are not blanket effects. Rather, they seem to manifest only under the most challenging and ecological listening scenarios in tasks that tap linguistic and cognitive processing (e.g., Swaminathan et al., 2015; Yoo and Bidelman, 2019).

Group differences in localization were smaller. Both cohorts localized targets (correct trials) within one to two speakers (i.e., <20–40 degrees), with slightly better performance in musicians (Figure 2C). One explanation for this more muted effect is that the localization task was delayed compared to recognition. There is evidence listeners can localize sound sources even if they cannot identify them (Rakerd et al., 1999). Determining *where* a signal is emitted in the soundscape has clear biological advantage over identifying *what* it is. It is also conceivable that musicians who play in an orchestra might have higher-level localization performance than those who play in a smaller ensemble. We did collect information on the *size* of musicians’ ensemble experience(s) to evaluate this possibility. However, supporting this notion, spatial tuning, and therefore localization abilities, does vary even among musicians depending on their relative position within an ensemble (e.g., conductor vs. player; Munte et al., 2001).

Musicians’ SIN benefits could result from both auditory and cognitive enhancements. From an auditory standpoint, musicians are more sensitive to basic perceptual attributes of sound including pitch, spectrotemporal features, and temporal fine structure, all critically important for normal and degraded speech perception (Kishon-Rabin et al., 2001; Micheyl et al., 2006; Bidelman and Krishnan, 2010; Bidelman et al., 2014a; Mishra et al., 2015; Madsen et al., 2017, 2019; Tarnowska et al., 2019). Moreover, physiological studies indicate that musicianship may enhance cochlear gain control via the olivocochlear efferent system (Bidelman et al., 2017), a pathway thought to provide an “antimasking” function to the inner ear (Guinan, 2006) that enhances signal in noise detection (Micheyl and Collet, 1996; Bidelman and Bhagat, 2015). However, we also found evidence for enhanced cognitive faculties in musicians (i.e., IQ and WM). IQ, WM, and attention presumably play a large role in SIN processing. Indeed, we found that WM was associated with better speech streaming and reduced target localization error at the cocktail party. Thus, musicians’ cocktail party benefits could reflect enhancements in domain-general cognitive abilities. Our findings parallel Schellenberg (2011) who found that musicianship was associated with IQ and Digit Span (WM and attention). They also converge with studies demonstrating relations between cognition (e.g., WM and auditory attention) and SIN performance in musical individuals (Strait and Kraus, 2011; Sares et al., 2018; Yoo and Bidelman, 2019; but see Escobar et al., 2020). Thus, musicians’ cocktail party benefits observed here might result from a refinement in both auditory-perceptual and cognitive abilities, both of which could aid degraded speech-listening skills. They might also result from musicians’ improved neural encoding of speech apparent at both brainstem and cortical levels (e.g., Parbery-Clark et al., 2009a; Bidelman et al., 2014b; Zendel et al., 2015; Mankel and Bidelman, 2018). Future electrophysiological studies are needed to evaluate the neural mechanisms underlying musicians’ improved cocktail party listening observed here.

Alternatively, musicians could have lower levels of internal noise, which would tend to aid cocktail party listening (Lufti et al., 2017). Given that our listeners were young, normal-hearing individuals, the locus of this noise would probably stem from group differences in central factors (e.g., lesser lapses in attention and higher WM), which can be considered their own form of internal noise. This interpretation is at least qualitatively supported by the superior WM we find in the music group (present study; Bugos et al., 2007; Bidelman et al., 2013; Yoo and Bidelman, 2019).

Links between listeners’ years of music training and (i) cocktail party recognition and (ii) cognitive measures (WM) suggest that musicians’ SIN benefits scale with experience. Interestingly, we found that listeners’ degree of music training predicted their QuickSIN performance even after controlling for WM. This suggests that musicianship might provide an additional boost to basic figure-ground speech perception beyond cognitive factors alone (e.g., Mankel and Bidelman, 2018; Yoo and Bidelman, 2019; but see Escobar et al., 2020). However, in contrast to the QuickSIN, the relation between musical training and *speech streaming* did not survive after controlling for WM. These results imply that while musicianship accounts for independent variance in simpler measures of SIN processing (i.e., QuickSIN), more complex SIN processing (i.e., cocktail party streaming) is driven more heavily by WM capacity. The degree to which listeners show successful speech/SIN processing likely represents a layering of inherent auditory listening skills (Mankel and Bidelman, 2018; Mankel et al., 2020), experience (Mankel and Bidelman, 2018), and cognitive factors including WM and attention (present study; Füllgrabe and Rosen, 2016; Oberfeld and Klöckner-Nowotny, 2016; Yoo and Bidelman, 2019). Our results are correlational in nature. Nevertheless, longitudinal (Torppa et al., 2018) and both quasi- and randomized-training studies in both younger and older adults (e.g., Kraus et al., 2014; Slater et al., 2015; Tierney et al., 2015; Zendel et al., 2019; Lo et al., 2020) provide converging evidence that musicianship causes gains in SIN processing in an experience-dependent manner.

Somewhat surprisingly, we did not find group differences in sustained attention, as measured via the SART, nor did attention correlate with cocktail party performance. These findings contrast with studies reporting attentional benefits in musicians (Strait et al., 2010; Thompson et al., 2017; Yoo and Bidelman, 2019) and work suggesting correlations between selective attention and individual differences in cocktail party listening (Oberfeld and Klöckner-Nowotny, 2016). Presumably, differences in results might be attributed to how attention is assessed. For example, selective attention, as measured via auditory backward masking (Strait et al., 2010; Yoo and Bidelman, 2019) and voice tracking (Madsen et al., 2019) paradigms, is superior in musicians. In contrast, we do not find group differences in *sustained* attention, as measured via the SART. Selective attention (Oberfeld and Klöckner-Nowotny, 2016), but not sustained attention (present study), correlates with cocktail party speech perception (but see Thompson et al., 2017). These studies suggest that the relation between attention and cocktail party listening varies with the specific (sub)construct of attention: selectively attending to a talker is arguably more relevant to parsing multi-talker mixtures than sustained, vigilance processes. Although not at ceiling performance, the relatively low error rates in the SART tasks (<30%) implies the lack of group effect might be due to the ease of the task. Moreover, the SART is a visual task. While there is some evidence that musicianship enhances visual processing (e.g., WM and multisensory binding) (George and Coch, 2011; Bidelman et al., 2013; Bidelman, 2016), visual attention may not differ between musicians and non-musicians (Strait et al., 2010). Nevertheless, in the cognitive domain of WM, we find a consistent musician boost in auditory mental capacity and strong links to SIN performance (e.g., Parbery-Clark et al., 2009b, 2011; Grassi et al., 2017; Yoo and Bidelman, 2019).

In conclusion, our findings confirm a relationship between musicianship and naturalistic cocktail party listening skills (stream segregation) but also suggest that cognitive factors may at least partially account for musicians’ SIN advantage. Nevertheless, the degree to which music experience causally improves cocktail party speech processing (e.g., see Kraus et al., 2014; Slater et al., 2015; Tierney et al., 2015; Zendel et al., 2019) or is governed by preexisting factors unrelated to formal music training (e.g., inherent auditory aptitude; Mankel and Bidelman, 2018; Bidelman and Mankel, 2019; Mankel et al., 2020) awaits empirical confirmation with the present cross-sectional data.

## Data Availability Statement

The raw data supporting the conclusions of this article will be made available by the authors, without undue reservation, to any qualified researcher. Requests for data and materials should be directed to GB.

## Ethics Statement

The studies involving human participants were reviewed and approved by the University of Memphis Institutional Review Board. The participants provided their written informed consent to participate in this study.

## Author Contributions

GB and JY designed the study, analyzed the data, and wrote the manuscript. JY collected the data. Both authors contributed to the article and approved the submitted version.

## Conflict of Interest

The authors declare that the research was conducted in the absence of any commercial or financial relationships that could be construed as a potential conflict of interest.

## Footnotes

1 The University of Memphis facility is a room-within-a room design featuring a 24 ft × 24 ft × 24 ft IAC anechoic chamber with floor/wall/ceiling Metadyne^®^ acoustic wedge coverage. The noise lock provides an STC 61 noise rating (low cutoff frequency = 100 Hz). A 36-channel Renkus-Heinz speaker array surrounds the seating location (16 were used in the experiment). Multichannel audio control is achieved by a TDT RX8 Multi-I/O Processor (Tucker Davis Technologies). Six Focusrite and Ashley Ne8250 amplifiers drive the speakers via a RedNet Dante MADI interface.

2 The QuickSIN uses four-talker babble and thus might be better related to streaming performance in the more comparable four-talker CRM condition. However, QuickSIN and CRM_4–talker_ performances were not correlated (*r* = −0.10; *p* = 0.60).
