# Supplementary material for: Musicians Show Improved Speech Segregation in Competitive, Multi-Talker Cocktail Party Scenarios
Source: Front Psychol. 2020 Aug 18;11:1927. doi: 10.3389/fpsyg.2020.01927 (PMC7461890; doi:10.3389/fpsyg.2020.01927)
Supplement: Supplementary file 1 [file Image_1.pdf]

Bidelman & Yoo - Supplemental Material

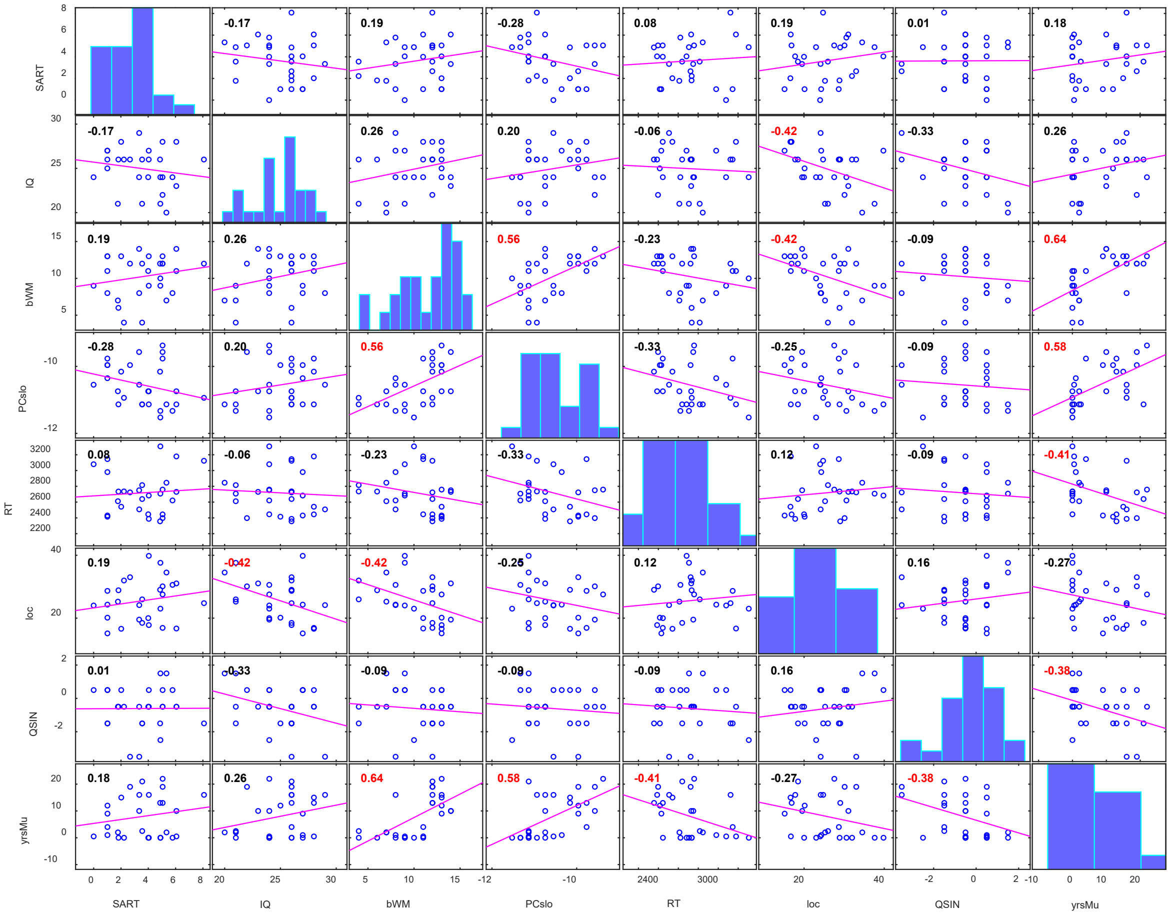

Figure S1: Bivariate correlations between pairwise tasks and musical training. Inset values denote Pearson's correlations. Red values = p<0.05 (uncorrected). SART=total errors in sustained attention; IQ=Raven's fluid IQ score; bWM=backward auditory working memory span; PCslope=change in %-accuracy with increasing maskers (see Fig. 2a, inset); RT=reaction time (collapsed across maskers); loc=localization error (collapsed across maskers); QSIN=QuickSIN reception thresholds; yrsMus= years of formal music training.
